# Continuity of Care and Lifestyle Intervention Programs for Spanish-Speaking Immigrants Without Health Insurance at a Free Clinic in Rhode Island

**DOI:** 10.5888/pcd21.240136

**Published:** 2024-11-21

**Authors:** Chilsea Wang, Jocelyn Yang, Julia Testa, Muneet Gill, Morgan Leonard, Anne S. De Groot

**Affiliations:** 1 Brown University School of Public Health, Providence, Rhode Island; 2 Clinica Esperanza/Hope Clinic, Providence, Rhode Island; 3 EpiVax, Inc, Providence, Rhode Island

## Abstract

**Introduction:**

We conducted a retrospective cohort study to evaluate changes in metabolic biomarkers among participants in Bridging the [Health Equity] Gap (BTG), a free program run by Clínica Esperanza/Hope Clinic (CEHC) for Spanish-speaking immigrants without health insurance in Rhode Island.

**Methods:**

From July 2019 through June 2021, 471 people volunteered to participate in the BTG program. Participants enrolled in lifestyle change classes and visited quarterly with health care providers. We reviewed medical records to collect data on blood glucose, total cholesterol, hemoglobin A_1c_ (HbA_1c_), and systolic and diastolic blood pressure at baseline and at 6, 12, 18, and 21 months after enrollment. We used paired *t* tests to identify changes in measurements and conducted a regression analysis to analyze trends in longitudinal patient outcomes.

**Results:**

From baseline to 6-month follow-up, we observed significant decreases in all participants’ mean HbA_1c_ (−0.71%), systolic (−5 mm Hg), and diastolic blood pressure (−2 mm Hg). At 12 months, significant decreases in mean HbA_1c_ persisted among participants with diabetes and prediabetes (−1.07%). At 12 months, participants with mean systolic blood pressure >120 mm Hg also had significant decreases in mean systolic blood pressure (−9 mm Hg), and patients with diastolic blood pressure >80 mm Hg had significant decreases in mean diastolic blood pressure (−9 mm Hg). Local population-level surges in COVID-19 due to Delta and Omicron variants were associated with increases in HbA_1c_ and blood glucose measurements above trendlines.

**Conclusion:**

The BTG program demonstrated resilience in supporting improvement in the metabolic biomarkers of participants, despite disruptions caused by the COVID-19 pandemic, the continued engagement of participants in self-care despite limited health care access, and underscores the positive role of free clinics among low-income, Spanish-speaking immigrants.

**Summary**

**What is already known on this topic?**

Lifestyle education programs can improve patient health and decrease use of emergency services, leading to savings for patients and health care systems. However, the effects of destabilizing factors (such as the COVID-19 pandemic) on access to care and health education programs have not been widely studied.

**What is added by this report?**

This report examines the resilience of a chronic disease management program in an uninsured, low-income Hispanic patient population from 2019 through 2022, during the COVID pandemic.

**What are the implications for public health practice?**

The results of this report support the implementation of lifestyle change programs to improve health outcomes during times of reduced access to care.

## Introduction

Inadequate health insurance coverage is a public health challenge in the US (1). Without the negotiation of health insurance providers, health care visits have substantially higher costs (2) and increased population reliance on preventable emergency care (3). Free clinics provide safety-net health care for populations that lack health insurance, some developing innovative health improvement programs or developing workforces that are linguistically and culturally tailored to their patient population (4). However, research is scant on the assessment of health outcomes during stressful periods, such as pandemics, in the population that uses free clinics.

Clínica Esperanza/Hope Clinic (CEHC) is a nonprofit free clinic for adults without health insurance in Providence, Rhode Island. More than 80% of CEHC patients speak primarily Spanish, and a large proportion are first-generation immigrants. CEHC patients face challenges as a result of poor health literacy and chronic health problems. The Bridging the [Health Equity] Gap (BTG) program was initiated at CEHC in 2015 with a mission to reduce health inequities among patients with chronic diseases through continuity of care, goal-setting appointments, and healthy lifestyle interventions. Program participants visit quarterly with CEHC health care providers and enroll in a healthy lifestyle intervention program, either *Vida Sana* or the National Diabetes Prevention Program (DPP). An evaluation of the financial and clinical effects of the BTG program before the pandemic is available elsewhere (4).

The objective of this study was to describe changes in 5 metabolic biomarkers among participants of the BTG program from July 2019 through July 2022. In this study, the COVID-19 pandemic enabled a natural stress-test of the resilience of the BTG program and improvements in the metabolic biomarkers of program participants. Our hypothesis was that BTG could support continual improvement in the metabolic biomarkers of program participants, despite disruptions caused by the COVID-19 pandemic. We expected to see a significant difference from the alternative (the null hypothesis) that metabolic biomarkers would not improve or change during participants’ enrollment in the BTG program.

## Methods

This retrospective cohort study was conducted in March 2024. We reviewed the medical records of 471 patients enrolled in BTG during regular clinical operations at CEHC. The evaluation period began on July 1, 2019, and ended on July 1, 2022.

### Participants

From October 1, 2019, through October 31, 2021, community health workers, who explained the nature and purpose of the BTG program, recruited participants from the main clinic of CEHC. Outreach efforts, such as those conducted at the Neighborhood Health Station (an outreach clinic opened during COVID-19), helped eligible participants find the main clinic to seek care and participation in BTG. Eligibility for BTG enrollment at CEHC was extended to all residents in Rhode Island who lacked health insurance and were living with diabetes or prediabetes, hypertension, cardiovascular disease, or overweight or obesity. Previous enrollees in the BTG program could also opt in to the new cohort. 

**Figure 1:**
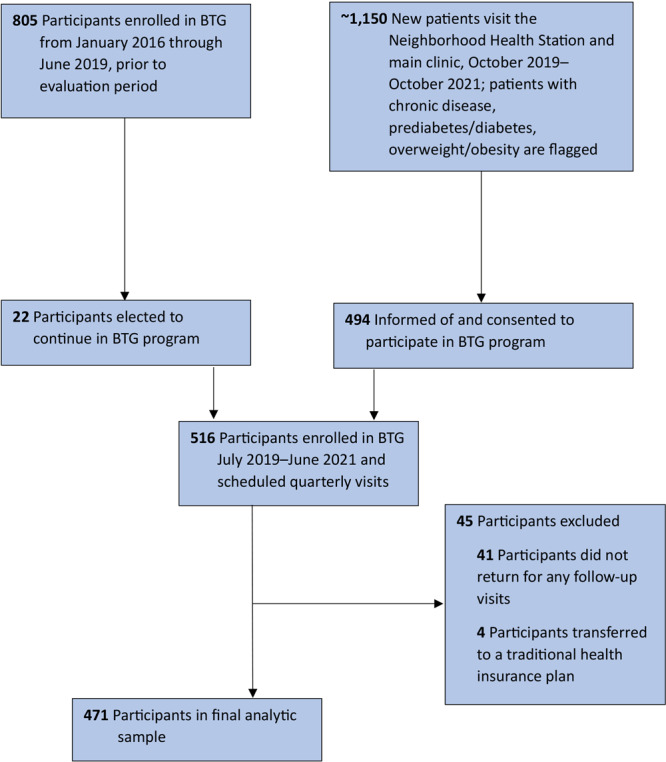
Flow of participants in the Clínica Esperanza/Hope Clinic Bridging the [Health Equity] Gap program, Providence, Rhode Island, 2019–2022.

Of the 805 participants enrolled in BTG from January 2016 through June 2019, 22 elected to join the new cohort. All participants signed (or signed again, if they were from the previous cohort) a partnership form giving CEHC permission to record individual data in a de-identified spreadsheet, documenting quarterly and yearly chronic disease assessments. Of 516 people recruited, 471 met enrollment criteria in the BTG program and had sufficient follow-up data (1 baseline measurement and at least 1 follow-up measurement) to be included in this evaluation (Figure 1). 

### Timeline

At enrollment in the BTG program (baseline), we measured the following metabolic biomarkers for each participant: blood glucose, HbA_1c_, total cholesterol, and systolic and diastolic blood pressure. At the conclusion of each visit, participants scheduled a follow-up appointment for 3 months later. We tracked each participant for a maximum of 8 calendar quarters after their enrollment date. Because the CEHC clinic population is transient as a result of fluctuating employment and documentation status, participants did not always schedule or attend a return visit. We called no-show participants once every calendar quarter until they either scheduled a follow-up or were discontinued from the study. If participants did not have a second set of measurements taken within 2 years after their baseline values, we excluded them from the dataset. We included metrics for all continuing participants in the longitudinal analysis until the date of their last follow-up visit. We determined instructional groups for our lifestyle programs based on start date or entry into the BTG program. We calculated the retention rate as a ratio of the number of participants who attended 1 or more follow-up visits to the total number of participants initially enrolled in the program.

### Lifestyle program

A locally developed lifestyle program (*Vida Sana*) involves classes taught to participants by using culturally attuned, linguistically appropriate materials, with teaching styles meeting the unique needs of participants’ low levels of health literacy (5). The course is taught by CEHC-trained multilingual, multicultural community health workers known as *Navegantes* (6). A pre-pandemic review of the *Vida Sana* program found significant improvements in blood pressure during the program (7,8).

CEHC also provides a formal DPP class to BTG participants with prediabetes. The objective of DPP is to reduce the risk of type 2 diabetes through a review of diet and physical activity (9). Participants receive structured, program-specific education on how to achieve and maintain lifestyle changes for 1 year. At CEHC, the course is taught in Spanish by *Navegantes*, who receive training from the Rhode Island Department of Health (6).

**Figure 2:**
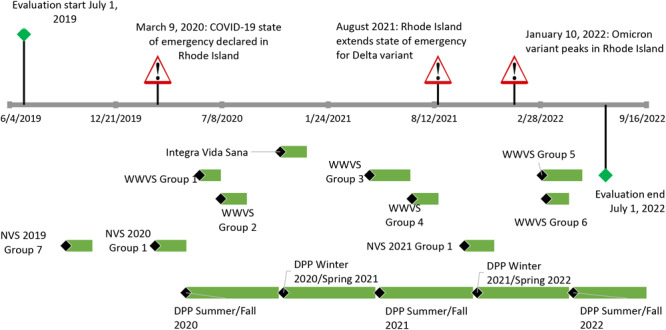
Timeline for the Clínica Esperanza/Hope Clinic Bridging the [Health Equity] Gap program in Providence, Rhode Island, for the evaluation period, July 1, 2019, to July 1, 2022. Names of programs reflect funding sources. Abbreviations: NVS, *Navegante Vida Sana*; WWVS, Wisewoman *Vida Sana.*

Individualized health coaching sessions in the *Vida Sana* and DPP formats were provided by *Navegantes *for patients whose schedules did not allow them to attend group classes or who needed to schedule a makeup class after missing a session of the group class. These sessions were charted as “One-on-Ones,” which were available to patients throughout the analysis period along with recurring cycles of *Vida Sana* and DPP classes (Figure 2). Participants were also invited to participate in repeated program sessions to maintain lifestyle changes and healthy habits.

**Table:** 

Date start	Date end	Duration, days	Label
**Classes**			
9/18/2019	11/6/2019	50	NVS 2019 Group 7
3/4/2020	5/2/2020	59	NVS 2020 Group 1
5/27/2020	7/22/2020	40	WWVS Group 1
7/6/2020	8/24/2020	49	WWVS Group 2
10/26/2020	12/14/2020	50	Integra Vida Sana
4/12/2021	6/28/2021	77	WWVS Group 3
7/1/2021	8/19/2021	50	WWVS Group 4
10/8/2021	12/3/2021	56	NVS 2021 Group 1
3/3/2022	5/18/2022	76	WWVS Group 5
3/11/2022	4/22/2022	43	WWVS Group 6
5/1/2020	10/31/2020	175	DPP Summer/Fall 2020
11/1/2020	4/30/2021	173	DPP Winter 2020/Spring 2021
5/1/2021	10/31/2021	176	DPP Summer/Fall 2021
11/1/2021	4/30/2022	173	DPP Winter 2021/Spring 2022
5/1/2022	10/31/2022	175	DPP Summer/Fall 2022
**Milestones**			
7/1/2019	—	—	Evaluation start July 1, 2019
3/9/2020	—	—	March 9, 2020: COVID-19 state of emergency declared in Rhode Island
8/19/2021	—	—	August 2021: Rhode Island extends state of emergency for Delta variant
1/10/2022	—	—	January 10, 2022: Omicron variant peaks in Rhode Island
7/1/2022	—	—	Evaluation ends July 1, 2022

### Data collection

This evaluation focused on patients enrolled from July 2019 through July 2021; outcomes were evaluated through July 2022.

Data were collected during normal clinic operations and later accessed for analysis by review of patient medical records. In addition to collecting data on metabolic biomarkers for each participant at baseline and at each subsequent clinic visit, we collected baseline data on participant sex (male or female), age, race and ethnicity, and height and weight. We calculated body mass index (BMI) as weight in kilograms divided by height in meters squared. We used adult BMI categories as specified by the Centers for Disease Control and Prevention: underweight (BMI <18.5), normal weight (BMI 18.5 to <25.0), overweight (BMI 25.0 to <30.0), and obese (BMI ≥30.0) (10).

We defined prediabetes as an HbA_1c_ of 5.7-6.4%, diabetes as HbA_1c_ ≥6.5%, hypertension as systolic blood pressure ≥130 mm Hg or diastolic blood pressure ≥80 mm Hg, and hyperlipidemia as total cholesterol ≥240 mg/dL.

We did not routinely collect data on income or educational level as part of this study; however, CEHC is situated in the Olneyville area of Providence and mostly serves patients from this neighborhood. The annual median family income of the Olneyville neighborhood is estimated at $23,200, based on weighted averages of census tracts and block groups from the 2010 census data and the 2010–2016 American Community Survey (11). In 2024, the federal poverty level for a family of 3 was $25,820, and for a family of 4 was $31,200 (12). CEHC has also been an awardee of the federal Community Development Block Grants program every year since 2015, and reports household income of its patients to the program in furtherance of its mission to help low-income people. Additionally, we surmise that the average educational level of most CEHC clinic patients is below high school level, and some are only able to read with difficulty (unpublished observation of A.D.G.).

### The COVID-19 pandemic and clinic operations

COVID-19 presented unique challenges to scheduling follow-up visits and providing the intervention. In the beginning of the pandemic (March 2020), all in-person visits were discontinued to reduce the chances of virus transmission at the clinic. After implementation of transmission prevention measures at the clinic, including previsit COVID-19 antigen testing and the installation of air filters in each examination room, we resumed in-person visits. We set up COVID-19 antigen testing and blood pressure tests at the Neighborhood Health Station to facilitate BTG visits; participants could also participate in *Vida Sana* or DPP group classes, or have One-on-One sessions with a *Navegante* at this location.

Class participation was flexible during the pandemic. With masking and testing, participants could attend in person. If participants were unable to attend group classes or when in-person classes were suspended during surges in COVID-19 rates, they could meet with *Navegantes* by video chat and in person for One-on-One visits to discuss goal setting, nutrition, and chronic disease management. Almost all lifestyle intervention programs, including *Vida Sana* and One-on-Ones, were conducted online in spring 2020 due to COVID restrictions, with DPP suspended, but by fall 2020, visits were in-person to maintain their effectiveness and attendance. Descriptions of the effect of COVID-19 on the uninsured Spanish-speaking population in Providence and the means by which CEHC provided access to free COVID-19 testing and vaccines for this population are described elsewhere (13,14).

### Statistical analysis

We organized participant counts in a symmetrical 4-term elliptical figure (a Venn diagram [15]). The 4 terms were the 4 risk factors: obesity/overweight, hypertension, hyperlipidemia, and diabetes/prediabetes.

We used paired *t* tests to identify significant changes in blood glucose, HbA_1c_, cholesterol, and systolic and diastolic blood pressure among participants overall. We also compared measurements that were matched by chronic condition: we examined measurements of HbA_1c_ and blood glucose among participants with prediabetes or diabetes, measurements of total cholesterol among participants with hyperlipidemia, and measurements of blood pressure among participants with hypertension. In addition, we compared baseline measurements between participants with at least 1 follow-up visit and participants who had only baseline measurements. We used the Analysis ToolPak feature in Excel version 16.62 (Microsoft Corp) for initial analysis and later verified with R version 4.4.0 (R Foundation for Statistical Computing), with the help of the packages ggplot2 (16) and OI-biostat (Dave Harrington, OI-biostat Labs; https://github.com/dave-harrington/oi_biostat_labs).

We calculated the mean HbA_1c_ and blood glucose levels of all participants in BTG for each calendar quarter from July 2019 through July 2022, for a total of 12 calendar quarters. Using these calculated means, we performed linear regression in R with the open-source ggplot package and subsequently added 95% CIs. We excluded outliers that resulted from surges caused by COVID-19 variants and used baseline and follow-up measurements in our calculation of means.

In a priori power analysis with G*Power version 3.1.9.7 (Heinrich-Heine-Universität Düsseldorf), the required sample size for significant results from a paired *t* test was 327 given a 2-sided significance level of .05 and an estimated effect size of 0.2, considered small for behavioral science (17). Two months after recruitment began, a meta-analysis of diabetes self-management education among Latino adults found a pooled effect size of −0.24 (±0.105 at 95% CI) for HbA_1c_ outcomes across 23 studies, validating our conservative estimate of effect size (18). G*Power estimated that the required sample size for an effect size of 0.24 with all other settings unchanged in the paired *t* test was 227. We conducted a sensitivity analysis with imputation based on a last-observation-carried-forward approach, followed by 2-sample *t* tests, which found no significant difference in participant outcomes at 6 and 12 months.

## Results

During the study period, 45 participants transferred to a traditional health insurance plan or were otherwise lost to follow-up. 

**Figure 3:**
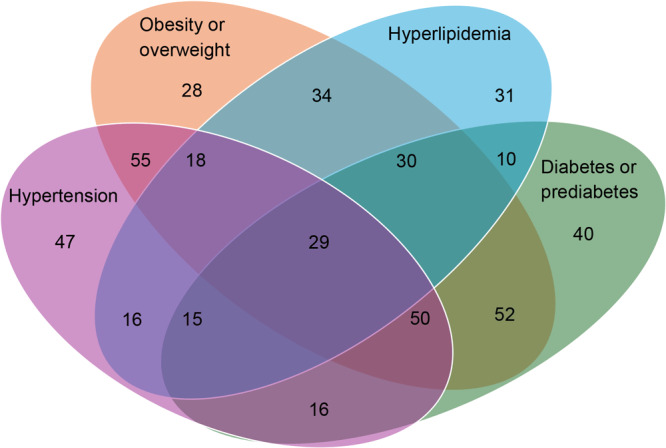
Venn diagram for the distribution of chronic conditions among participants in the Clínica Esperanza/Hope Clinic Bridging the [Health Equity] Gap program in Providence, Rhode Island, for the evaluation period, July 1, 2019, to July 1, 2022. All values are number of participants.

Of 471 participants, 211 (44.7%) were women and 260 (55.2%) were men. Most participants self-identified as Hispanic or Latino (91%). The mean (IQR) age was 50 (40–59). Moreover, 52.7% of participants lived with hypertension, 51.8% lived with diabetes or prediabetes, 39.2% lived with hyperlipidemia, and 62.5% lived with overweight or obesity; 69.6% of participants lived with 2 or more of these conditions, and 30.4% of participants lived with 3 or more. For example, 29 (6.1%) had hyperlipidemia, diabetes or prediabetes, obesity or overweight, and hypertension (Figure 3). Overall, the program retention rate was 91%. 

**Table:** 

Condition	No. of participants
Obesity/overweight only	28
Obesity/overweight + hypertension	55
Hypertension only	47
Hyperlipidemia only	31
Hyperlipidemia + obese/overweight	34
Hyperlipidemia + obese/overweight + hypertension	18
Hyperlipidemia + hypertension	16
Hyperlipidemia + diabetes/prediabetes	10
Hyperlipidemia + diabetes/prediabetes + obesity/overweight	30
Hyperlipidemia + diabetes/prediabetes + obesity/overweight + hypertension	29
Hyperlipidemia + diabetes/prediabetes + hypertension	15
Diabetes/prediabetes only	40
Diabetes/prediabetes + obesity/overweight	52
Diabetes/prediabetes + obesity/overweight + hypertension	50
Diabetes/prediabetes + hypertension	16

Evaluation at 6, 12, 18, and 21 months showed that mean blood glucose levels among participants with diabetes or prediabetes declined between −45.8 and −136.0 points, mean HbA_1c_ among participants with diabetes or prediabetes declined between −0.43 and −1.17 points, and the blood pressure of participants with hypertension declined by −2 to −11 mm Hg (systolic) and −1 to −9 mm Hg (diastolic) (Table).

**Table:** Paired *t* Tests for Differences in Means Among Participants in the Clínica Esperanza/Hope Clinic Bridging the [Health Equity] Gap Program, Providence, Rhode Island, July 2019-July 2021^a^

Measurement	Months from baseline	No. of participants	Baseline mean (SD)	Follow-up mean (SD)	Difference in means	*P* value^b^
**HbA_1c_, %**						
All participants	0	386	6.58 (2.19)	—	—	—
	6	96	7.17 (2.71)	6.46 (1.68)	−0.71	.005
	12	56	6.61 (2.42)	6.28 (1.73)	−0.33	.21
	18	23	6.28 (1.86)	6.75 (2.12)	0.47	.27
	21	14	6.29 (1.65)	6.02 (0.72)	−0.27	.38
Participants with diabetes (HbA_1c_ ≥6.5%) or prediabetes (HbA_1c_ 5.7%–6.4%)	0	242	7.46 (2.49)	—	—	—
	6	55	8.18 (2.99)	7.01 (1.94)	−1.17	<.001
	12	26	8.10 (2.90)	7.03 (2.23)	−1.07	.002
	18	13	7.24 (2.64)	6.78 (1.80)	−0.46	.21
	21	8	6.79 (1.82)	6.36 (0.79)	−0.43	.44
**Blood glucose, mg/dL**						
All participants	0	347	139.5 (82.4)	—	—	—
	6	92	153.3 (102.2)	139.2 (65.6)	-14.05	.27
	12	60	150.8 (93.7)	126.9 (53.2)	-23.93	.09
	18	22	169.9 (114.9)	147.4 (97.7)	-22.5	.49
	21	14	200.4 (143.4)	107.3 (23.6)	-93.07	.03
Participants with diabetes (HbA_1c_ ≥6.5%) or prediabetes (HbA_1c_ 5.7%–6.4%)	0	242	141 (84)	—	—	—
	6	31	206 (130)	160 (77.6)	−45.8	.02
	12	12	242 (121)	189 (85.9)	−53.2	.13
	18	5	308 (125)	233 (82.3)	−75.0	.15
	21	3	244 (111)	108 (22.6)	−136.0	.12
**Total cholesterol, mg/dL**						
Participants with hyperlipidemia (≥240 mg/dL)	0	183	237.2 (31.2)	—	—	—
	6	34	204.6 (47.1)	204.6 (47.2)	0	.99
	12	25	224.7 (54.7)	221.6 (72.2)	−3.1	.78
	18	4	204.3 (53.7)	184.5 (42.5)	−19.8	.54
	21	6	221.3 (50.9)	226.3 (29.1)	5.0	.82
**Systolic blood pressure, mm Hg**						
All participants	0	469	133.2 (22.0)	—	—	—
	6	118	135 (21.3)	130 (19.9)	−5	.02
	12	86	134 (22.0)	129 (15.6)	−5	.02
	18	28	132 (25.1)	124 (18.2)	−8	.11
	21	17	136 (21.4)	134 (21.6)	−2	.58
Participants with hypertension (systolic blood pressure >120 mm Hg)	0	246	140 (19.0)	—	—	—
	6	56	144 (19.5)	137 (17.2)	−7	.01
	12	45	144 (20.5)	135 (13.6)	−9	.006
	18	15	143 (28.2)	132 (18.6)	−11	.23
	21	13	142 (21.0)	140 (21.3)	−2	.80
**Diastolic blood pressure, mm Hg**						
All participants	0	469	83 (13.3)	—	—	—
	6	118	82 (13.0)	80 (11.6)	−2	.04
	12	86	83 (12.1)	79 (9.9)	−4	.01
	18	28	82 (13.2)	78 (10.7)	−4	.16
	21	17	85 (11.4)	77 (12.5)	−8	.02
Participants with hypertension (diastolic blood pressure >80 mm Hg)	0	223	94 (10.6)	—	—	—
	6	13	89 (14.7)	88 (10.3)	−1	.90
	12	10	88 (6.33)	79 (7.8)	−9	.01
	18	5	84 (11.2)	76 (3.6)	−8	.28
	21	6	83 (8.0)	81 (17.1)	−2	.85

Abbreviation: —, does not apply; HbA_1c_, glycosylated hemoglobin A_1c_.

a Means in this table were calculated based on months elapsed from baseline, for patients enrolled between 2019 and 2021, within the data evaluation window of 2019 to 2022.

b Determined by *t* test; *P* < .05 considered significant.

The evaluation of participant outcomes as a function of their time spent in the BTG program demonstrates improvement at the 1-year mark, with results diminishing after that point. We found significant improvements in metabolic biomarkers at 6 and 12 months, but none at 18-month follow-up and only one at 21-month follow-up (Table). The average HbA_1c_ declined significantly among all BTG participants from baseline to 6 months (from 7.17% to 6.46%; *P* = .005) and among BTG participants with diabetes or prediabetes at 6 months (8.18% to 7.01%; *P* < .001) and 12 months (8.10% to 7.03%; *P* = .002) (Table). In addition, we found a significant decline in blood glucose among participants with diabetes or prediabetes at 6 months, in systolic blood pressure among all participants and participants with hypertension at 6 and 12 months, in diastolic blood pressure among all participants at 6, 12, and 21 months, and among participants with hypertension at 12 months. We also found a significant decline in blood glucose from baseline among all patients who remained in the program at 21 months (n = 14). We found no other significant changes.

In the comparison of participants with at least 1 follow-up visit and participants who had only baseline measurements, participants lost to follow-up did not have significantly different measurements of blood pressure, HbA_1c_, or blood glucose levels at baseline, but they did tend to have significantly higher total cholesterol levels (Appendix).

**Figure 4:**
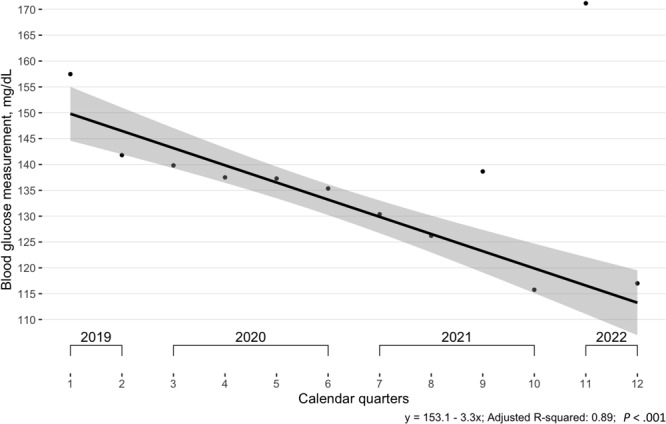
Mean blood glucose measurements among participants (N = 471) in the Clínica Esperanza/Hope Clinic Bridging the [Health Equity] Gap Program, Providence, Rhode Island, July 2019–July 2022. All visits (baseline and follow-up) were used in calculation of means. The regression excluded outliers found during the Delta (August 2021) and Omicron (January 2022) waves of COVID-19. Shading indicates 95% CIs.

**Figure 5:**
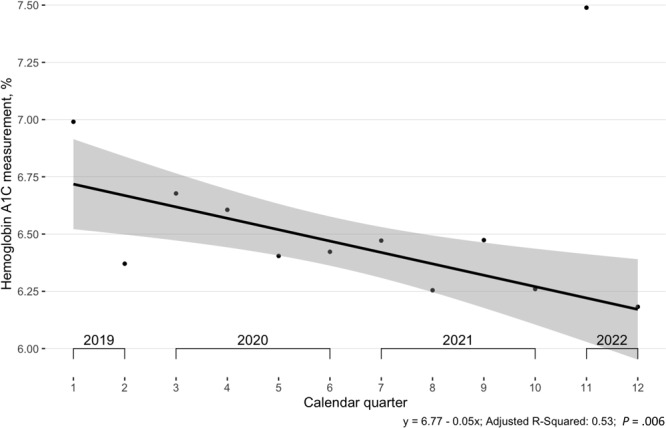
Mean glycosylated hemoglobin A_1c_ (HbA_1c_) measurements among participants (N = 471) in the Clínica Esperanza/Hope Clinic Bridging the [Health Equity] Gap Program, Providence, Rhode Island, July 2019–July 2022. All visits (baseline and follow-up) were used in calculation of means. The regression excluded the outlier found during the Omicron (January 2022) wave of COVID-19. Shading indicates 95% CIs.

In a comparison of mean blood glucose in each calendar quarter in the overall BTG cohort, the trendline had a significantly negative slope (*R* ^2^ = 0.89) that was, however, interrupted during periods of high rates of COVID-19 transmission (Figure 4). This finding was corroborated by mean HbA_1c_, which also had a significantly negative slope but had a worse fit, with an adjusted *R* ^2^ of 0.53 and outliers in the same calendar quarters (Figure 5).

## Discussion

From 2018 through 2022, the percentage of Rhode Island residents without health insurance ranged from 4.8% to 4.9% despite introduction of low-cost health insurance for residents and state expansion of Medicaid (2,19). This statistic obscures racial and ethnic disparities in health insurance coverage. Hispanic residents of Rhode Island are more likely than non-Hispanic residents to lack health insurance, have no regular primary care physician, and experience financial barriers to seeking care (14). The proportion of non-Hispanic White residents without health insurance decreased from 5.2% in 2018 to 1.5% in 2022, while the proportion of Hispanic residents without health insurance was higher overall and decreased from 24.3% to 21.0% during the same period (19,20).

CEHC was established in 2008 to address the need for primary and preventive health care for people without health insurance, with a focus on culturally competent care for all — and the BTG program aligns with this mission. Most BTG participants in our study cohort self-identified as Hispanic or Latino (91%), similar to the 2016–2017 cohort, in which nearly all identified as non-White Hispanic or Latino (4), and in contrast to the general population of Rhode Island, at 17% Hispanic or Latino (21,22). This difference may be due to the location of CEHC in Olneyville, a neighborhood in which 63.9% of the population is Hispanic (23). 

Hispanic people are more likely than non-Hispanic White people in Rhode Island to have diabetes, hypertension, and cardiovascular disease. In 2010, the prevalence of diabetes in the state was almost 2 times higher in the Hispanic population (13%) than in the non-Hispanic White population (6.7%) (21). Hypertension, a major contributor to cardiovascular disease, also disproportionately affects Hispanic people compared with non-Hispanic White people (24). Although evidence is conflicting on cardiovascular disease death rates in the Hispanic population relative to the non-Hispanic White population, the rates of nonfatal myocardial infarction and loss of disability-adjusted life years are higher in the Hispanic population than in the non-Hispanic White population in the US (24,25). These trends are not unique to the Hispanic population in the US, with Black, American Indian, Alaska Native, Native Hawaiian, and Pacific Islander populations also facing similar disparities (26). New interventions to prevent complications from poorly managed chronic disease should focus on these groups.

In 2020, rates of primary care and emergency department use decreased across the nation before slowly rising in demand, even above pre-pandemic levels (27,28). Use of the BTG program mirrored this trend, with sharply decreased numbers of office visits in the early pandemic months and subsequent increases. The BTG cohort in this study consisted of 471 patients during the 24-month enrollment window from July 2019 to July 2021, a rate of 19.6 patients per month. This rate is similar to previously published BTG enrollment rates at CEHC’s main clinic in 2018: 805 patients during 41 months (19.6 patients per month) (4). The overall similarity in enrollment rates, despite an initial dip in participation for this study, may have resulted from increased community trust in the BTG program when the clinic began establishing other services, such as free COVID-19 testing, vaccination, and Paxlovid distribution. These services may have raised awareness of CEHC programs.

Participation in BTG activities by women was negatively and disproportionately affected by surges in COVID-19. Overall, 44.7% of BTG participants were women and 55.2% were men. This disproportion may have resulted from increased childcare responsibility caused by closures of childcare facilities and restrictions on children visiting CEHC’s main clinic during the pandemic. Addressing childcare needs may be critical to improving health care access for parents of young children.

Improving access to care for people without health insurance may decrease associated costs imposed on the health care system (29). The potential benefits of BTG participation to the local health system are notable. Our previous study found that BTG participants had 61% fewer potentially preventable emergency department visits, resulting in an average annual potential savings to the Rhode Island health care system of $781,122 (4). Better control of chronic diseases through managing blood glucose, normalizing HbA_1c_, and reducing blood pressure has been estimated at $1,445 to $2,073 per patient (30).

A survey of low-income Hispanic adults with diabetes at clinics in the Southwest and Midwest found that during the pandemic, many Hispanic adults were unable to receive medical care for diabetes and had an increased frequency of hyperglycemia (31). Barriers to health care during the pandemic, such as job-related pressures, transportation needs, and childcare considerations, also affected BTG participants. No-show rates were high for postbaseline office visits, especially in months where new strains of COVID-19 were emerging in Rhode Island. However, despite this, retention in the program improved significantly to 91%, compared with the previous iteration, where 26.1% were lost to follow-up or transferred to other providers (4). This unexpected result could be attributed to telehealth, which has been shown to increase appointment attendance by reducing barriers related to cost and time, particularly benefiting patients who live far from medical providers (32–34). It is also possible that the current iteration of the program increased its outreach and fostered more timely re-engagement for all CEHC patients as a result of increased funding for vaccinations and COVID-19 testing.

Across metabolic biomarkers, when we compared patient outcomes at different calendar quarters, we found that most significant improvements occurred during the 2019–2020 period (before the COVID-19 pandemic). During the 2020–2021 period (peak COVID-19), biomarkers also improved from baseline, but with a smaller magnitude, reflecting observed decreases in the number of participants and smaller changes in metabolic values, potentially related to disruptions in clinic schedules and patient attitudes toward clinic access. The Delta surge was officially recognized by the state of Rhode Island in August 2021 (35), followed by the Omicron wave, reported by local news organizations in January 2022 (36). These surges coincided with poorer outcomes in blood glucose levels, HbA_1c_ values, and blood pressure control among study participants. Total cholesterol did not change significantly during the evaluation period, possibly because of the known preference of CEHC health care providers to encourage lifestyle changes first, followed by statin medications.

### Limitations

Our study has several potential limitations. First, interpretation of the results may be limited because the cohort was not randomized and the BTG program involved voluntary participation (by motivated participants), which precluded an intent-to-treat analysis. Second, we did not collect information about participants’ level of education, which may be a confounder of metabolic outcomes. Third, the data can be generalized only to patients who are willing and able to engage with health education such as that provided by the BTG program, reflecting a potential selection bias such that participants included in this evaluation had the means and will to return for at least 1 follow-up visit. However, sensitivity analysis showed that participants who dropped out appeared to have similar baseline characteristics as those who remained in the study, except for total cholesterol levels, and adding those participants back into data based on their last observation carried forward showed no change in significance. In addition, all BTG participants had a metabolic comorbidity, which does not perfectly reflect real-world populations.

### Conclusion

Lack of access to health care contributes to underdiagnosis and undertreatment of chronic diseases and poor continuity of care. Hospitalization of patients without health insurance also has negative financial consequences for patients and health care systems. Innovative programs such as BTG and the associated *Vida Sana* program, tailored to the cultural and linguistic preferences of their communities, are needed to improve access to care populations that lack health insurance and have low literacy levels. Our results reject the null hypothesis. Despite the disruptions of the COVID-19 pandemic, BTG supported continual improvement in participants’ metabolic biomarkers. Our study illustrates that access to free health care, continuity of care, and lifestyle education programs have positive effects on the health of people who lack health insurance. With our collective efforts, neighborhood by neighborhood, we may yet bridge the gaping divide of health care disparity.

## Appendix Supplemental Table

**Table:** Comparison of Baseline Measurements Between Participants Lost to Follow-Up and Participants Included in Analysis, by Sex, Year of Data Collection, and Type of Measurement (Baseline or Follow-Up), the Clínica Esperanza/Hope Clinic Bridging the [Health Equity] Gap Program, Providence, Rhode Island, July 2019-July 2022

Metabolic indicator	Patients lost to follow-up, mean (SD)	Participants with 1 or more follow-up visits, mean (SD)	*P* value^a^
Systolic blood pressure, mm Hg	137.1 (22.3)	134.5 (22)	.10
Diastolic blood pressure, mm Hg	82.7 (12.9)	83.7 (13.6)	.20
Hemoglobin A_1c_, %	6.48 (2.1)	6.696 (2.3)	.18
Blood glucose, mg/dL	144.0 (87.9)	136.0 (78.2)	.18
Body mass index, kg/m^2^	31.2 (5.3)	31.6 (5.6)	.18
Total cholesterol, mg/dL	213.0 (5.6)	202.0 (43.1)	.01

Abbreviation: HbA_1c_, glycosylated hemoglobin A_1c_.

^a^ Determined by *t* test; *P* < .05 considered significant.
